# Lung Ultrasound for Imaging of B-Lines in Dogs and Cats—A Prospective Study Investigating Agreement between Three Types of Transducers and the Accuracy in Diagnosing Cardiogenic Pulmonary Edema, Pneumonia and Lung Neoplasia

**DOI:** 10.3390/ani11113279

**Published:** 2021-11-16

**Authors:** Andrzej Łobaczewski, Michał Czopowicz, Agata Moroz, Marcin Mickiewicz, Marta Stabińska, Hanna Petelicka, Tadeusz Frymus, Olga Szaluś-Jordanow

**Affiliations:** 1 Veterinary Clinic Auxilium, Arkadiusz Olkowski, Królewska Street 64, 05-822 Milanówek, Poland; alobaczewski007@gmail.com; 2 Division of Veterinary Epidemiology and Economics, Institute of Veterinary Medicine, Warsaw University of Life Sciences-SGGW, Nowoursynowska Street 159, 02-776 Warsaw, Poland; michal_czopowicz@sggw.edu.pl (M.C.); agata_moroz@sggw.edu.pl (A.M.); marcin_mickiewicz@sggw.edu.pl (M.M.); 3 Private Veterinary Clinic, Wyszyńskiego Street 12/11, 41-200 Sosnowiec, Poland; marta_stabinska@yahoo.co.uk; 4 Veterinary Clinic Peteliccy, 1 Maja 27, 96-300 Żyrardów, Poland; h.petelicka@gmail.com; 5 Department of Small Animal Diseases with Clinic, Institute of Veterinary Medicine, Warsaw University of Life Sciences-SGGW, Nowoursynowska Street 159, 02-776 Warsaw, Poland; tadeusz_frymus@sggw.edu.pl

**Keywords:** agreement beyond chance, linear transducer, lung ultrasonography, microconvex, phased array transducer, small animals, thorax

## Abstract

**Simple Summary:**

The high usefulness of thorax ultrasound examination in diagnosing lower respiratory tract diseases is well-evidenced in both human and veterinary medicine. However, the type of transducer (microconvex, linear, and phased array) which is most effective in the examination of dogs and cats remains unknown. In this study we examined dogs and cats using the three types of transducers and we assessed how consistent they were in detecting and quantifying B-lines. Moreover, we developed a method that allowed to quantitatively evaluate the occurrence of B-lines in cardiogenic pulmonary edema (CPE), pneumonia and lung neoplasm, referred to as the lung ultrasound score (LUS_score_). Our results indicate that microconvex and linear transducers can be used interchangeably. LUS_score_ may help distinguish between lung neoplasms (lower values) and CPE or pneumonia (higher values) in dogs, and between CPE (higher values) and pneumonia or lung neoplasms (lower values) in cats.

**Abstract:**

Transthoracic heart and lung ultrasound (LUS) was performed in 200 dogs and cats with dyspnea to evaluate the agreement between the results obtained using three types of transducers (microconvex, linear, and phased array) and to determine the accuracy of LUS in discriminating between three conditions commonly causing dyspnea in companion animals: cardiogenic pulmonary edema (CPE), pneumonia, and lung neoplasm. The agreement beyond chance was assessed using the weighted Cohen’s kappa coefficient (κ_w_). The highest values of κ_w_ (>0.9) were observed for the pair of microconvex and linear transducers. To quantify B-lines the lung ultrasound score (LUS_score_) was developed as a sum of points describing the occurrence of B-lines for each of 8 standardized thoracic locations. The accuracy of LUS_score_ was determined using the area under ROC curve (AUROC). In dogs AUROC of LUS_score_ was 75.9% (CI 95%: 65.0% to 86.8%) for distinguishing between lung neoplasms and the two other causes of dyspnea. In cats AUROC of LUS_score_ was 83.6% (CI 95%: 75.2% to 92.0%) for distinguishing between CPE and the two other causes of dyspnea. The study shows that results obtained with microconvex and linear transducers are highly consistent and these two transducers can be used interchangeably. Moreover, the LUS_score_ may help identify dogs with lung neoplasms and cats with CPE, however its diagnostic accuracy is only fair to moderate.

## 1. Introduction

Lung ultrasound (LUS) examination is an easy and useful diagnostic tool for lower respiratory tract pathology. It is recommended in the suspect of: cardiogenic pulmonary edema (CPE), consolidation, atelectasis, embolism, neoplasia, pneumonia, pneumothorax, interstitial lung diseases especially with fibrosis, as well as in any other lower respiratory tract signs like dyspnea, pleural pain or fluid and acute cough [1]. This technique is relatively inexpensive, non-ionizing, portable, and easily available. In addition, it does not require anesthesia or special patient positions causing discomfort, which is very important in critical care units.

Several special rapid examination protocols have been developed for animals in emergency situations: Veterinary Bedside lung ultrasound exam (Vet BLUE) and Thoracic Focused Assessment with Sonography for Trauma (TFAST) [2,3]. They allow to establish a tentative or even definitive diagnosis very quickly, usually within less than 3 min. This accelerates the diagnosis and allows to initiate the treatment earlier which can be critical for saving the animal’s life. This complementary examination should be interpreted with reference to the patient’s medical history and clinical manifestation.

Basic ultrasound examination of the thorax is able to recognize artefacts like: pulmonary-pleural line, A- and B-lines. The pulmonary-pleural line is a horizontal, hyperechoic line, moving forward and back with ventilation, and seen below the rib line. A-lines are artifactual repetitions of the pulmonary-pleural line displayed at regular intervals deeper to the pleura as bright, horizontal lines. They are mainly visible in normal, aerated lungs. In contrast, B-lines (also called ultrasound lung rockets) are visible in interstitial-alveolar edema. They result from many backward and forward movements of ultrasound beams between air and fluid, generating a long, vertical hyperechoic artefact, beginning on the pleural line and stretching down the screen, moving along with the pleural line synchronously with respiration [4]. They arise due to the accumulation of small amounts of fluid in the lung tissue surrounded by air which creates a high impedance gradient. Their number and width correlate with the intensity of the pathology. However, B-lines are insufficient for the definitive diagnosis, as they result from interstitial-alveolar fluid, which can occur both in cardiogenic and non-cardiogenic pulmonary edema, as well as in acute respiratory distress syndrome, pulmonary hemorrhage of various etiologies, pneumonia, lung contusion, neoplastic metastasis to the lung or pulmonary fibrosis [5,6]. It is important to assess the number of B-lines since more lines in the follow-up examination will indicate progression of the disease, and less will confirm that the treatment is effective [6,7,8]. A single B-line may be physiological, however more indicate lung pathology. The aforementioned artefacts are easy-to-learn which makes basic LUS highly reproducible—the agreement between examiners in B-line detection varies from 92% to 97% depending on their experience [9,10].

In veterinary medicine microconvex and linear transducers are currently recommended in LUS [5,8]. In the human medicine, there are no specific prior requirements. It has been long believed that microconvex or phased arrays transducers are preferable, mainly due to their small head which makes the examination technically easier. They are also better at visualizing deeper structures, however the pleural line is usually less visible [11]. Therefore, a linear transducer is recommended when imaging of the pleural line in of most importance. The first recommendations regarding minimal equipment for LUS included a basic ultrasound device equipped with either microconvex or phased array transducer with the frequency of 2.0–5.0 MHz [12]. Over the last decade, the recommendations have changed and the type of transducers depends on the hospital departments they are used in. In critical care units, using a 5.0 MHz microconvex probe is usually recommended [10]. Nowadays, ultrasound machines are usually by default equipped with all three types of transducers. This allows to examine both in a critical situation (when there is no time for switching the transducer) and to perform routine LUS in stable patients. When the patient is stable, microconvex, phased array and linear transducers are recommended to get the best quality image or detect as many details as possible, which is impossible with examination with using only one probe [1].

Our study aimed to evaluate: (1) the agreement between three types of transducers (microconvex, linear and phased array) in imaging of B-lines in dogs and cats with CPE, pneumonia and lung neoplasm; (2) the accuracy of LUS in distinguishing between CPE, pneumonia, and lung neoplasms.

## 2. Materials and Methods

According to the Polish legal regulations (The Act of the Polish Parliament of 15 January 2015 on the Protection of Animals Used for Scientific or Educational Purposes, Journal of Laws 2015, item 266), the consent of the ethics committee is not required for this type of research and no consent from the ethical committee is required for postmortem tissue use. All owners were informed about specific purpose of the study and the standard veterinary practice to examine patients using 2–3 types of ultrasound probes to obtain the best quality image or more details not visible with a single probe. All owners provided consent for the inclusion of their animals in the study. No animal was euthanized for the purpose of the above study. Animals that were euthanized at the request of the owners in order to withdraw from persistent therapy since they were diagnosed with malignant neoplasm, in a severe clinical condition, and did not respond to the treatment.

### 2.1. Animals

The study was carried out between January 2016 and July 2019 in two veterinary clinics located in Poland. Two hundred dogs and cats with dyspnea (the respiratory rate increased above 30 breaths/min at rest) were prospectively enrolled. In each patient basic demographic and medical data were recorded (species, breed, gender, age, body weight, history of disease, medications applied during last month), a complete clinical examination was performed (including heart and respiratory rates, presence of cardiac murmur and abnormal respiratory sounds), and followed by the radiography and ultrasound examination of the chest. On this basis an initial diagnosis was made and a relevant therapy was started. Patients were only included in the further analysis if B-lines were detected in LUS.

### 2.2. Ultrasound Examination and Classification of Patients

**Figure 1:**
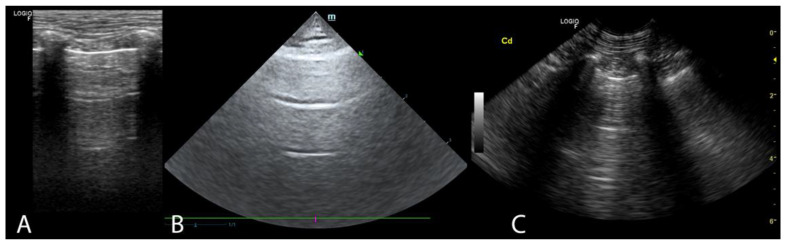
A-lines. (**A**). Linear probe (**B**). Phased array probe (**C**). Convex probe.

**Figure 2:**
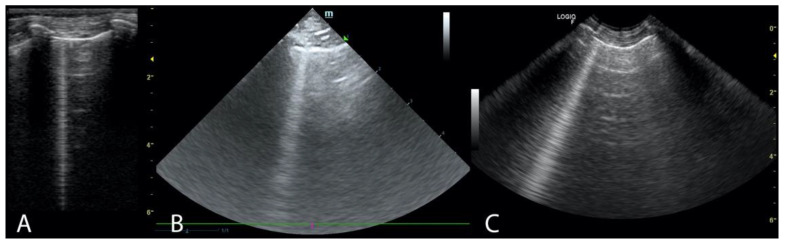
Single B-line (**A**). Linear probe (**B**). Phased array probe (**C**). Convex probe.

**Figure 3:**
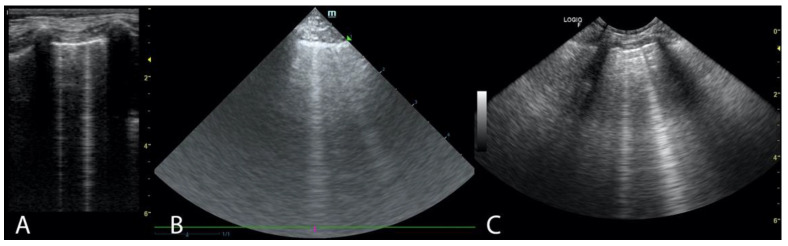
Two B-lines. (**A**). Linear probe (**B**). Phased array probe (**C**). Convex probe.

**Figure 4:**
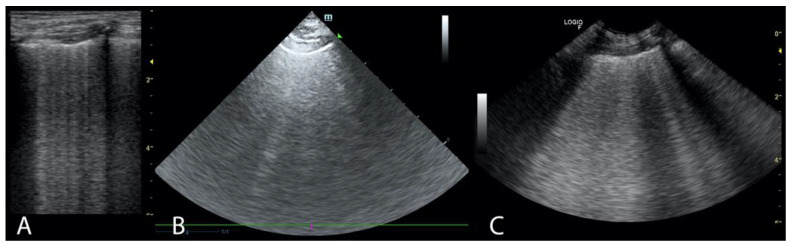
Numerous discernible B-lines (**A**). Linear probe (**B**). Phased array probe (**C**). Convex probe.

**Figure 5:**
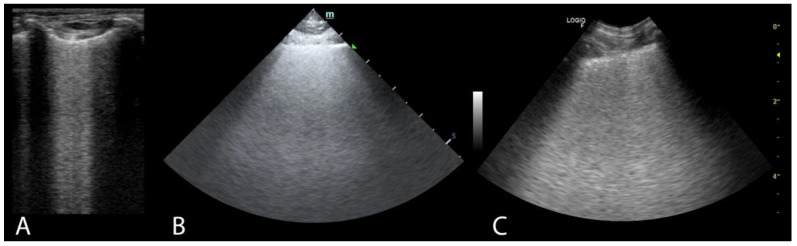
Numerous indiscernible B-lines (**A**). Linear probe (**B**). Phased array probe (**C**). Convex probe.

Two ultrasound devices were used: Mindray M7 with 4–2 s MHz phased array transducer and a L14-6ns MHz linear transducer, and GE Healthcare Logiq F6 with L6-12-RS MHz linear transducer and a 8C-RS MHz microconvex transducer. The imaging depth was set at 3–6 cm depending on the size of the animal and the focus position was set as close to the pleural line as possible. All examinations were performed in standing or sternal positions without clipping (hair was parted), after application of an appropriate amount of alcohol and coupling gel with the transducer placed directly on the chest. All transducers were positioned transverse to the ribs in order to visualize the “gator sign” (the pleural line and two ribs). Four regions were examined on each thoracic side (caudodorsal, perihilar, middle, and cranial) with one scan for each region, according to the Vet BLUE protocol [6,13]. The presence of A-lines with a lung sliding was considered to signify a physiological aerated lung. The interstitial-alveolar edema was recognized by the presence of B-lines. Additional ultrasound abnormalities were described according to Ward et al. [2] following the descriptions used in human medicine as the shred sign, tissue-like sign or nodule sign [1]. The shred sign is a manifestation of partial lung consolidation. The deeper border of the consolidated lung tissue connected with the aerated lung is shredded and irregular. The tissue-like sign is when the lung resembles the liver tissue and it results from translobar consolidation. The nodule sign is circumscribed, completely surrounded by the aerated lung. B-lines extend from the distal border of each type of consolidation downwards the screen [2]. The occurrence of B-lines was described according to the 5-point scale: no B lines—0 points (Figure 1), a single B-line—1 point (Figure 2), double B-line—2 points (Figure 3), numerous discernible B-lines—3 points (Figure 4), and numerous indiscernible B-lines—4 points (Figure 5).

Ultrasonographic examinations were performed by two board-certified specialists in thoracic ultrasound (O.S.-J. and A.Ł.), each with 10-year experience in this field. A.Ł. used a GE Healthcare Logiq F6, and O.S.-J. a Mindray M7 device. Examinations with different transducers were performed immediately one after another at intervals shorter than 5 min. In order to place subsequent transducers to the same thoracic regions, they were applied to 8 places previously covered with gel. The B-lines were counted over a single intercostal space according to Lisciandro [14]. In all patients, echocardiographic examination was also performed to rule out congenital or acquired heart defects, heart tumors, pericardial effusion and other cardiac diseases. It included right parasternal long and short axis view and left parasternal views with apical four-chamber and five-chamber views. The views were mainly aimed at obtaining measurements of the left atrium (LA), aorta (Ao), left and right ventricles, the left ventricular wall thickness (LVWT) as well as left and right ventricular outflow tract velocities.

The advanced myxomatous valvular degeneration (MVD) was considered highly probable when the left ventricular internal dimension in diastole (LVIDd) to Ao ratio (LVIDd/Ao) was >3 and LA/Ao was >2.4 [15,16].

Dilated cardiomyopathy (DCM) in dogs was diagnosed if the following abnormalities were found [14,16,17]:

- left ventricular M-mode systolic or diastolic dimensions exceeding reference values for the given body weight,
- LA/Ao > 2.1,
- fractional shortening (FS) < 20%,
- left ventricular ejection fraction (LVEF) < 40

Myocarditis was confirmed by histopathological and microbiological examination [18].

Hypertrophic cardiomyopathy (HCM) in cats was diagnosed if: LVWT was >10 mm in diastole and La/Ao was >2.0 [14,16,17].

Restrictive cardiomyopathy (RCM) was diagnosed in cats if the dimensions of the LV walls and of the left chamber were in reference range and marked dilatation of the atria was detected.

DCM was diagnosed in cats when dilatation of the four cardiac cavities was visualized [16,17].

A preliminary diagnosis was made immediately after thoracic radiography and ultrasonographic examination. All lung scans and cine clips were saved and anonymized for further analysis to avoid the confounding effect resulting from the use of different transducers. Detailed analysis of all cine clips was performed after the examination had been completed by both examiners.

The clinical condition of each animal was monitored until its condition considerably improved or the animal died, and the sonographic diagnosis was confronted with the response to the treatment or autopsy findings, respectively. On this basis the patient was assigned to one of four diagnostic groups: cardiogenic pulmonary edema (CPE), pneumonia, lung neoplasm, or others. CPE was diagnosed based on chest radiography and the presence of B-lines combined with correct, smooth image of the pleural line in patients with advanced heart disease found in the echocardiographic examination. Good response to diuretic and oxygen therapy (disappearance of B-lines within 24 h and decrease of respiratory rate to <30 per minute) was considered to confirm the diagnosis of CPE.

**Figure 6:**
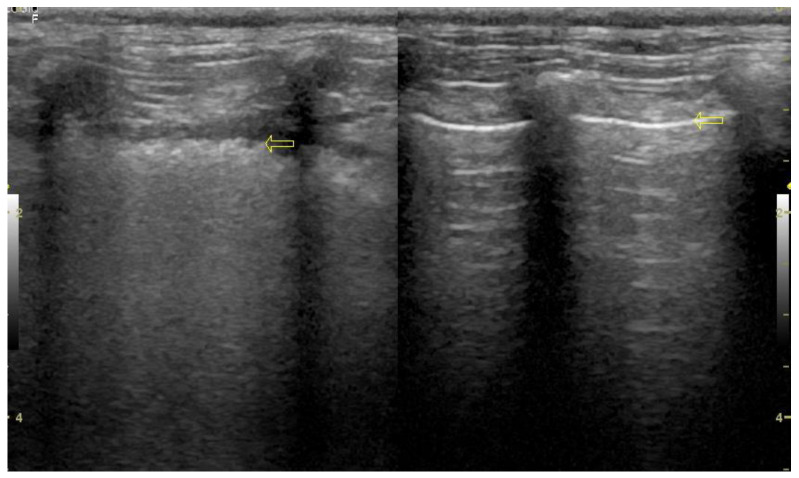
Thickened pulmonary-pleural line (yellow arrow on the left), for comparison normal pleural line (yellow arrow on the right).

In all patients with suspected pneumonia clinical examination, blood test, LUS and chest radiography were performed. In all animals dyspnea was observed. B-lines accompanied by subpleural consolidations or irregularly thickened, blurred pleural in ultrasound examination suggested pneumonia (Figure 6). Broad spectrum antibiotics were administered for at least 2 weeks. After 2 weeks, a follow-up LUS was performed and if lung sliding and A-lines (normal filled with air lung image) were visualized and patient’s condition reverted to normal the patient was diagnosed with pneumonia. According to Hew (2016), clinical improvement after treatment confirmed the diagnosis [19]. It is because patient-reported outcome is one of the main indicators of the treatment efficacy, since the main objective of medical care is to increase patient well-being.

**Figure 7:**
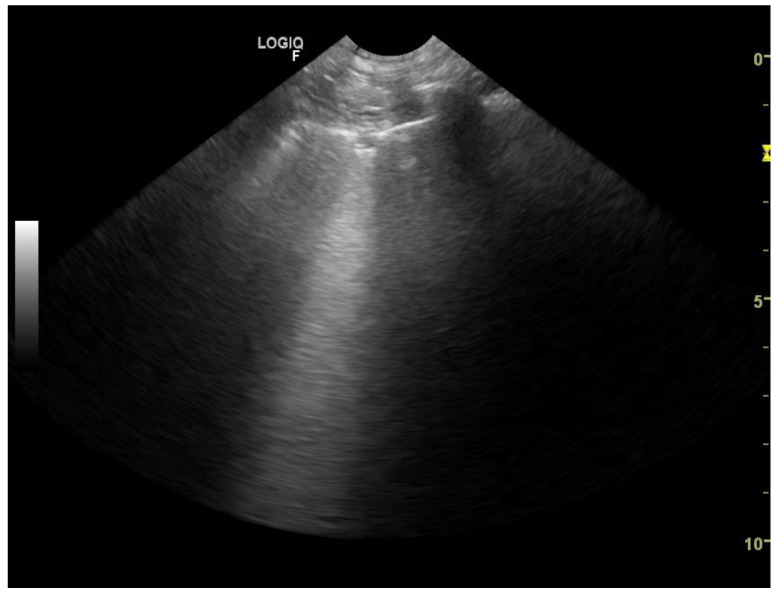
Single subpleural consolidation (nodule type)—convex probe.

**Figure 8:**
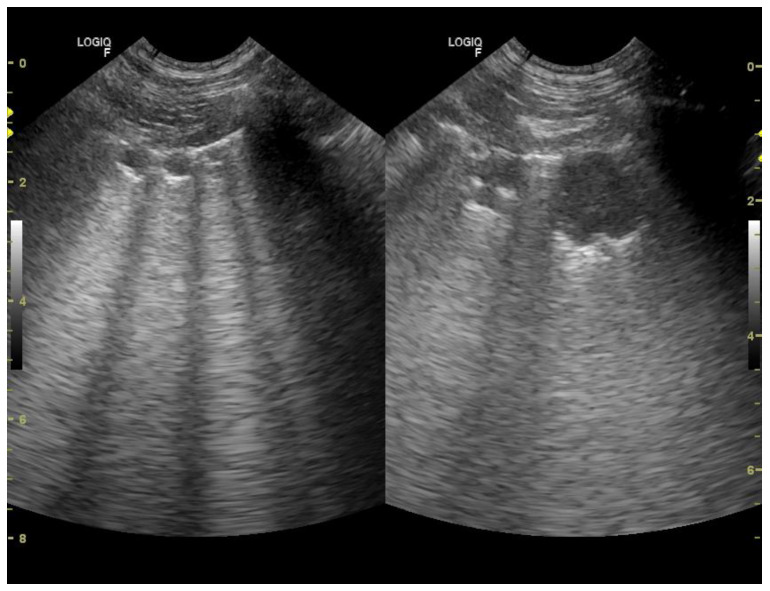
Numerous subpleural consolidators (nodule type)—convex probe.

**Figure 9:**
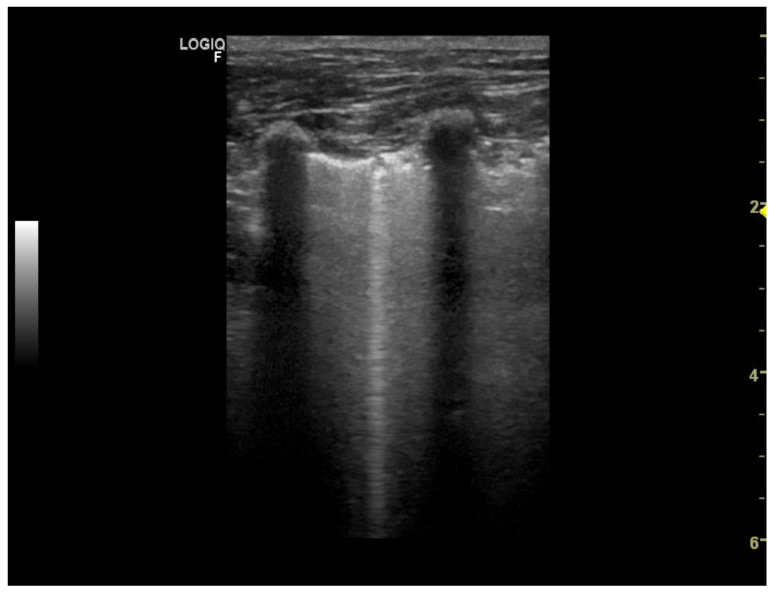
Single subpleural consolidation (nodule type)—linear probe.

Patients suspected of having neoplastic lesions underwent chest radiography (48 patients) or chest CT-scan (5 patients) together with LUS. To confirm the neoplastic disease, the patients underwent ultrasound-guided transthoracic fine needle aspiration lung biopsy (25 patient) or, if euthanized, anatomopathological and histopathological examination of the lung (10 patients). The remaining 18 patients had been diagnosed with primary malignant neoplasm, confirmed by histopathological examination within preceding 12 months. During the visit with dyspnea chest radiographs showed uniform in density, with smooth, well-margined borders round nodules. LUS showed subpleural nodulus (Figure 7, Figure 8 and Figure 9). Treatment with broad-spectrum antibiotics and steroids was ineffective.

### 2.3. Statistical Analysis

The agreement beyond chance between three types of ultrasound transducers (microconvex, linear [two linear probes were treated as one type], and phased array) at 8 standardized locations (caudodorsal, perihilar, middle, and cranial lung lobe on each side) was assessed using the weighted Cohen’s kappa (κ_w_), with 95% confidence interval (CI 95%) calculated according to the formula of Fleiss [20]. Weights were set as numbers between 1 and 0 so that distances between categories reflected the importance of the difference between results, with the weight of 1 for fully consistent results and 0 for the most distant results (Table 1). Values of κ_w_ of ≤0.40 indicated low, 0.41–0.60—moderate, 0.61–0.80—high, 0.81–0.90—very high, and >0.90—almost perfect agreement. Agreement beyond chance was determined for the entire study population as well as separately for dogs and cats.

Then, the 5-point classification of the occurrence of B-lines was used for developing the numerical score (lung ultrasound score, LUS_score_) combining the number of B lines with the locations in which they were observed. LUS_score_ was calculated as the sum of points (*n*) at each of 8 standardized location (i), on the basis of the following equation: ${\mathrm{LUS}}_{\mathrm{score}}=\sum_{i=1}^{8}n_{i}$. Given that *n* = {0,1,2,3,4}, and i = {1,2,3,4,5,6,7,8}, LUS_score_ could take integer values from 0 to 32 points.

In the agreement analysis the LUS_score_ was presented as the arithmetic mean and standard deviation (±SD), and compared between the three transducers using the paired-sample Student’s t-test and the line of equality plot. The magnitude of differences between the LUS_score_ measurements expected in 95% of examinations was demonstrated for the three pairs of ultrasound transducers using the Bland-Altman limits of agreement (LoAs), for the entire study population as well as separately for patients with three main pathological conditions: CPE, pneumonia, and lung neoplasm.

In the accuracy analysis the LUS_score_ was presented as the median, interquartile range (IQR), and range, and compared between animal species and three main pathological conditions using the Mann-Whitney U test and the Kruskal-Wallis H test, respectively. Accuracy was presented as the area under receiver operating characteristic (ROC) curve (AUROC) and assessed as follows: >90%—a highly accurate test, >80% to 90%—a moderately accurate test, >70% to 80%—a fairly accurate test, ≤70%—a poorly accurate test [21,22]. Then, the optimal cut-off value was determined using the maximum Youden’s index (J) criterion. and diagnostic sensitivity (Se), specificity (Sp) and positive/negative likelihood ratio (LR+/−) were computed at the optimal cut-off value. Proportions were compared between groups using the Pearson’s χ^2^ test and CI 95% for proportions were calculated using the Wilson score method. A significance level (α) was set at 0.05. Statistical analysis was performed in TIBCO Statistica 13.3 (TIBCO Software Inc., Palo Alto, CA, USA).

## 3. Results

### 3.1. Animals

Two hundred animals with dyspnea were enrolled in the study (Table S1)—116 dogs (58.0%; 57 males) and 84 cats (42.0%; 52 males). The age of the dogs ranged from 3 months to 17 years with the median (IQR) of 11 (8 to 13) years. Thirty three dogs (28.4%) were cross-breeds. The most common breed was Yorkshire terrier (*n* = 14, 16.7% of 83 pedigree dogs), followed by dachshunds and French bulldogs (each *n* = 6, 7.2% of pedigree dogs). The age of the cats ranged from 3 months to 21 years with the median (IQR) of 10 (5 to 13) years. Most of the cats were domestic shorthair (*n* = 64, 76.2%), followed by Maine coons (*n* = 7, 8.3% of pedigree cats).

Echocardiography revealed cardiac disease in 35 dogs (30.2%) and 34 cats (40.5%). Among dogs 25 had MVD (71.5% of dogs with heart disease), 4 (11.4%) had both MVD and tricuspid valve disease (TVD), 4 (11.4%) had DCM, and 2 dogs (5.7%) had myocardial hypertrophy due to inflammation. Among cats 17 (50.0%) had HCM, 14 (41.2%) had DCM (two of them with concurrent with TVD), and 3 cats (8.8%) had RCM.

CPE was diagnosed in 35 dogs (30.2%) and 34 cats (40.5%), pneumonia in 41 dogs (35.3%) and 22 cats (26.2%), and lung neoplasm in 37 dogs (31.9%) and only 16 cats (19.1%). In 15 animals other conditions were recognized: in 3 dogs (2.6%) and 9 cats (10.7%) intrathoracic fluid was the only finding, and 3 cats (3.6%) had pneumothorax.

Neither the distribution of gender (*p* = 0.141) nor age (*p* = 0.134) differed significantly between the diagnoses.

### 3.2. Agreement between Results Obtained by Different USG Transducers

#### 3.2.1. B-Lines

One hundred seventy one animals (93 dogs and 78 cats) which were examined using at least 2 different type of transducers (including 54 patients with all 3 transducers) were included in the agreement analysis.

The agreement between results obtained by the microconvex and linear transducers was almost perfect (κ_w_ > 0.90), regardless of the thoracic region where they were placed (Table 2), both in dogs (Table 3) and cats (Table 4). The agreement was lower although still high (κ_w_ between 0.61 and 0.80) to very high (κ_w_ between 0.81 and 0.90) when any of the two transducers was compared to the phased array transducer. In this case the agreement seemed to be the lowest at perihilar and middle locations, also both in dogs and cats.

The analysis of LUS_score_ confirmed a very high agreement between results obtained by microconvex and linear transducers—expected difference in 95% of measurements of LUS_score_ ranged by ±2 points. The agreement between microconvex or linear and phased array transducers was lower—the expected difference in 95% of measurements of LUS_score_ varied by ±5–6 points (Table 5). This discrepancy was evident in patients with pulmonary edema and pneumonia, but almost unapparent in animals with lung neoplasms (Table 6).

#### 3.2.2. A-Lines

Unpublished sonographic (Vet BLUE) data from additional 20 cats and 31 dogs with lung sliding and A-lines visualized in all regions by both ultrasound specialists were used as negative control.

### 3.3. Accuracy of LUS_score_ in 3 Main Conditions Causing Dyspnea

One hundred eighty five animals (113 dogs and 72 cats) with CPE, pneumonia or lung neoplasia were included in the accuracy analysis. Fifteen patients without B-lines in sonography, animals with incomplete history or clinical data as well as those that could not be followed up long enough after the sonographic diagnosis were excluded from the study.

**Figure 10:**
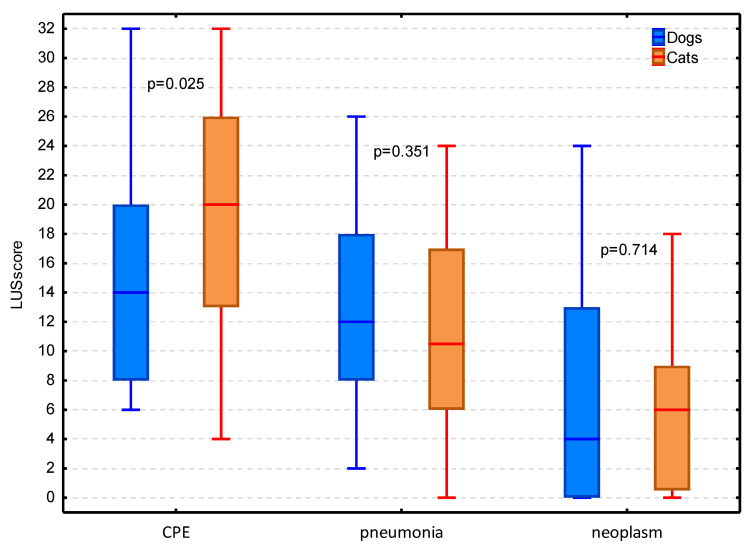
The lung ultrasound score (LUS_score_) in three main conditions causing dyspnea in dogs and cats given as the median, IQR and range. CPE stands for cardiogenic pulmonary edema.

In dogs LUS_score_ was significantly lower in lung neoplasms (median (IQR) of 4 (0–13)) than in CPE (median (IQR) of 14 (8–20); *p* < 0.001) and pneumonia (median (IQR) of 12 (8–18); *p* = 0.001), whereas it did not differ significantly between the latter two conditions (*p* = 0.999). In cats LUS_score_ was significantly higher in CPE (median (IQR) of 20 (13–26)) than in pneumonia (median (IQR) of 10 (6–17); *p* = 0.018) and lung neoplasms (median (IQR) of 6 (0–10); *p* < 0.001), whereas it did not differ significantly between the latter two conditions (*p* = 0.141). The LUS_score_ was significantly higher in cats than in dogs with CPE (*p* = 0.025). In the two other conditions causing dyspnea the LUS_score_ did not differ significantly between dogs and cats (Figure 10).

In dogs AUROC of LUS_score_ when used to distinguish between lung neoplasms and other causes of dyspnea (i.e., CPE and pneumonia) was 75.9% (CI 95%: 65.0% to 86.8%; *p* < 0.001) which corresponded to fair diagnostic accuracy. At an optimal cut-off value of 5 it had Se of 93.7% (CI 95%: 86.0%, 97.3%) and Sp of 62.2% (CI 95%: 46.1%, 75.9%), which corresponded to LR+ of 2.48 (CI 95%: 1.63, 3.76) and LR- of 0.10 (CI 95%: 0.04, 0.25).

In cats AUROC of LUS_score_ when used to distinguish between CPE and other causes of dyspnea (pneumonia and lung neoplasm) was 83.6% (CI 95%: 75.2% to 92.0%; *p* < 0.001), which corresponded to moderate diagnostic accuracy. At an optimal cut-off value of 11 it had Se of 82.4% (CI 95%: 66.5%, 91.7%) and Sp of 70.0% (CI 95%: 56.2%, 80.9%), which corresponded to LR+ of 2.75 (CI 95%: 1.75, 4.31) and LR- of 0.25 (CI 95%: 0.12, 0.53).

## 4. Discussion

LUS is relatively easy to perform and interpret, especially if analyzing basic features, such as normal air-filled lung, pulmonary edema, neoplasm, and free pleural fluid. However, the results highly depend on the skills and experience of the investigator. In the human medicine, basic skills necessary to be competent in chest ultrasound examination have been defined [23]. Unfortunately, no such standards in veterinary medicine have so far been developed. Moreover, guidelines indicating the most recommended types of transducers are lacking in the veterinary medicine.

B-lines are observed mainly in pulmonary edema, pneumonia or lung neoplasm. In pulmonary edema, the pleural line is smooth, whereas in pneumonia or neoplasm it is thickened and subpleural consolidations are visible (shred, tissue or nodule signs). These lesions accompanied by the presence of the B-lines strongly suggest non-cardiogenic pulmonary edema. Additionally, cardiogenic B-lines are mainly on both sides of the thorax, more diffuse and disappear after a few hours of diuretic therapy [2,24]. B-lines alone both in humans and animals clearly indicate pulmonary edema. B-lines with shred or tissue signs are visible mainly in pneumonia, and the nodule sign mainly in neoplasia. Ultrasound imaging can visualize only subpleural lesions, however, in the human medicine it is estimated that 90–99% of the lesions are in this location [25].

To our knowledge, this is the first study analyzing the agreement of results obtained by the three basic types of ultrasound transducers in the detection and quantitative evaluation of B-lines in small animals. It showed that the scans obtained with the use of microconvex and linear transducers were very highly consistent (kappa coefficient > 90%), while the agreement between any of them and the phased array transducer was moderate to high (κ_w_ between 60% and 90%). This observation applies both to dogs and cats. The agreement of results obtained by the microconvex and linear transducers is similarly high in all four quadrants in which the transducer is placed. It is of great importance as the available equipment varies considerably between veterinary clinics. In the pursuit of minimalizing the costs some of them search for a single transducer which will let them examine as many organs as possible. The agreement between results obtained using any of the aforementioned transducers and the phased array transducer is not only lower but also varies between scanning locations. Generally, it is the highest in the caudodorsal region and the lowest in perihilar and middle region.

In our study we introduced the concept of a numerical score which would allow to express the results of LUS in a quantitative manner. Our concept, referred to as LUS_score_, is a simple sum of points corresponding to the number of B-lines observed in each of eight standardized locations of the transducer. Similar concept has recently been presented by Ward et al. (2019) [2] however their score assigned very high number of points to the situation in which the number of B-lines was infinite. This created a wide gap between the two neighboring assessments—more than three but still countable number of B lines scored 4, and infinite number of B-lines scored 10. As a result, the distribution of quantitative results was likely to be considerably right-hand skewed and it added much weight to the picture of “wet lung”. Our LUS_score_ combines any situation in which the number of B-lines exceeds 3 in one category, which simplifies interpretation, especially for less experienced examiners working on the lower-quality ultrasound devices. Our LUS_score_ is based on equal point-distances between the categories which corresponds to the ratio measure scale. Thanks to that, we can use LUS_score_ for numerical comparison of the severity of sonographic signs between two patients as well as between two time points in one patient (follow-up examinations)—twofold decrease in LUS_score_ indicates twofold improvement in the lung picture while in the Ward’s score (2019) the change from the category of infinite number of B-lines in all quadrants (8 × 10 points = 80 points) to the category of >3 but countable number of B-lines in all quadrants (8 × 4 points = 32 points) results in more than twofold drop of the score while the improvement is in our opinion negligible or slight at most.

Our proposal is also different from the concept of Ward et al. (2018) [8] which is based on counting of the number of positive quadrants, which in our opinion is still more a categorical assessment than quantifying of the condition’s severity. Moreover, it allows only 9 categories of results (0 through 8 positive quadrants) and requires one strict dichotomous criterion allowing to class the quadrant as positive or negative be arbitrarily assumed.

Having developed a method enabling quantification of LUS signs we decided to evaluate its accuracy in identifying three conditions causing dyspnea which are most often diagnosed using LUS-CPE, pneumonia and lung neoplasm. Interestingly, LUS_score_ in CPE turned out to be significantly higher in cats than in dogs. As a consequence, the LUS_score_ could to some extent help to distinguish between cardiogenic and non-cardiogenic conditions in cats, however its diagnostic performance proved to be only moderate, even worse than reported by Ward et al. (2019). In dogs the LUS_score_ in CPE did not differ significantly from values in pneumonia. Nevertheless, in this species the LUS_score_ allowed to identify patients with neoplastic disease, which had significantly lower values than those with CPE or pneumonia although its accuracy was only fair.

## 5. Conclusions

The highest agreement of qualitative and quantitative results of LUS was observed using the microconvex and linear transducer which indicates that they can be used interchangeably. Therefore, these two transducers should be recommended in order to increase repeatability and reproducibility of LUS results.

The LUS_score_ proposed in this paper may help distinguish between lung neoplasms (values < 5) and CPE or pneumonia (values ≥ 5) in dogs, and between CPE (values ≥ 11) and pneumonia or lung neoplasms (values < 11) in cats, however its accuracy is only fair to moderate.

## Figures and Tables

**Table 1:** Weights used in pairwise comparisons.

5-Point Classification of the Occurrence of B-Lines	0	1	2	3	4
0	1	0.9	0.5	0.1	0
1	0.9	1	0.9	0.5	0.1
2	0.5	0.9	1	0.9	0.5
3	0.1	0.5	0.9	1	0.9
4	0	0.1	0.5	0.9	1

**Table 2:** Weighted Cohen’s kappa (κ_w_) with CI 95% for pairwise comparisons of three types of ultrasound transducers at 8 standardized scanning locations (4 on each side) in the entire study population.

Pair of Transducers Compared	Microconvex vs. Linear (*n* = 150)		Microconvex vs. Phased Array (*n* = 55)		Linear vs. Phased Array (*n* = 74)	
Standardized location of the transducers	Right side	Left side	Right side	Left side	Right side	Left side
caudodorsal	1.00 (1.00, 1.00)	0.993 (0.984, 1.00)	0.930 (0.876, 0.983)	0.947 (0.909, 0.986)	0.942 (0.900, 0.984)	0.941 (0.895, 0.986)
perihilar	0.991 (0.980, 1.00)	0.973 (0.951, 0.995)	0.798 (0.666, 0.930)	0.771 (0.636, 0.906)	0.854 (0.757, 0.952)	0.830 (0.723, 0.937)
middle	0.972 (0.954, 0.990)	0.988 (0.975, 1.00)	0.771 (0.630, 0.911)	0.828 (0.715, 0.940)	0.770 (0.654, 0.886)	0.844 (0.753, 0.935)
cranial	0.981 (0.963, 0.999)	0.991 (0.981, 1.00)	0.895 (0.814, 0.975)	0.915 (0.851, 0.979)	0.886 (0.799, 0.973)	0.929 (0.878, 0.979)

**Table 3:** Weighted Cohen’s kappa (κ_w_) with CI 95% for pairwise comparisons of three ultrasound transducers at 8 standardized locations (4 on each side) in dogs.

Pair of Transducers Compared	Microconvex vs. Linear (*n* = 76)		Microconvex vs. Phased Array (*n* = 33)		Linear vs. Phased Array (*n* = 48)	
Standardized location of the transducers	Right side	Left side	Right side	Left side	Right side	Left side
caudodorsal	1.00 (1.00, 1.00)	0.993 (0.978, 1.00)	0.904 (0.815, 0.994)	0.953 (0.903, 1.00)	0.924 (0.859, 0.989)	0.932 (0.860, 1.00)
perihilar	0.994 (0.981, 1.00)	0.967 (0.938, 0.995)	0.828 (0.654, 1.00)	0.767 (0.584, 0.949)	0.886 (0.767, 1.00)	0.846 (0.707, 0.986)
middle	0.969 (0.943, 0.996)	0.988 (0.971, 1.00)	0.815 (0.619, 1.00)	0.904 (0.786, 1.00)	0.786 (0.630, 0.943)	0.899 (0.802, 0.995)
cranial	0.983 (0.964, 1.00)	0.989 (0.973, 1.00)	0.875 (0.751, 999)	0.961 (0.916, 1.00)	0.857 (0.727, 0.988)	0.963 (0.927, 0.999)

**Table 4:** Weighted Cohen’s kappa (κ_w_) with CI 95% for pairwise comparisons of three ultrasound transducers at 8 standardized locations (4 on each side) in cats.

Pair of Transducers Compared	Microconvex vs. Linear (*n* = 74)		Microconvex vs. Phased Array (*n* = 22)		Linear vs. Phased Array (*n* = 26)	
Standardized location of the transducers	Right side	Left side	Right side	Left side	Right side	Left side
caudodorsal	1.00 (1.00, 1.00)	0.994 (0.981, 1.00)	0.962 (0.914, 1.00)	0.939 (0.878, 1.00)	0.968 (0.927, 1.00)	0.950 (0.899, 1.00)
perihilar	0.987 (0.970, 1.00)	0.978 (0.946, 1.00)	0.756 (0.553, 0.959)	0.774 (0.571, 0.977)	0.799 (0.629, 0.970)	0.796 (0.622, 0.970)
middle	0.974 (0.948, 0.999)	0.987 (0.969, 1.00)	0.687 (0.483, 0.891)	0.708 (0.497, 0.918)	0.736 (0.569, 0.903)	0.739 (0.557, 0.922)
cranial	0.979 (0.947, 1.00)	0.994 (0.981, 1.00)	0.922 (0.849, 0.995)	0.831 (0.690, 0.972)	0.935 (0.871, 0.998)	0.868 (0.745, 0.990)

**Table 5:** The agreement between the lung ultrasound score (LUS_score_) calculated using three types of transducers for the entire study population.

Pair of Transducers.	No. of Pairs	Mean (±SD) Difference (CI 95%)	*p*-Value	Limits of Agreement (CI 95%)	
				Lower	Upper
Microconvex and linear	150	−0.09 ± 0.64 (−0.19, 0.02)	0.102	−1.3 (−1.5, −1.2)	1.2 (1.0, 1.4)
Microconvex and phased array	55	0.71 ± 2.92 (−0.08, 1.50)	0.078	−5.0 (−6.4, −3.7)	6.4 (5.1, 7.8)
Linear and phased array	74	0.73 ± 2.71 (0.10, 1.36)	0.023	−4.6 (−5.7, −3.5)	6.0 (5.0, 7.1)

**Table 6:** The agreement between the lung ultrasound score (LUS_score_) calculated using three types of transducers for dogs and cats with 3 main diseases.

Pair of Transducers	No. of Pairs	Mean (SD) Difference	*p*-Value	Limits of Agreement (CI 95%)	
				Lower	Upper
Patients with cardiogenic pulmonary edema (*n* = 69)					
Microconvex and linear	43	−0.07 ± 0.86 (−0.33, 0.19)	0.596	−1.7 (−2.2, −1.3)	1.6 (1.2, 2.1)
Microconvex and phased array	17	0.29 ± 3.58 (−1.55, 2.14)	0.739	−6.7 (−9.9, −3.5)	7.3 (4.1, 10.5)
Linear and phased array	33	0.58 ± 2.93 (−0.46, 1.61)	0.267	−5.2 (−7.0, −5.2)	6.3 (4.5, 8.1)
Patients with pneumonia (*n* = 63)					
Microconvex and linear	59	−0.14 ± 0.66 (−0.31, 0.04)	0.117	−1.4 (−1.7, −1.1)	1.1 (0.9, 1.4)
Microconvex and phased array	24	1.33 ± 3.10 (0.02, 2.64)	0.046	−4.8 (−7.0, −2.5)	7.4 (5.2, 9.7)
Linear and phased array	27	1.19 ± 3.06 (−0.03, 2.40)	0.055	−4.8 (−6.9, −2.7)	7.2 (5.1, 9.3)
Patients with lung neoplasm (*n* = 53)					
Microconvex and linear	36	−0.06 ± 0.41 (−0.19, 0.08)	0.422	−0.9 (−1.1, −0.6)	0.7 (0.5, 1.0)
Microconvex and phased array	12	0.00 ± 1.13 (−0.72, 0.72)	0.999	−2.2 (−3.5, −1.0)	2.2 (1.0, 3.5)
Linear and phased array	12	0.08 ± 0.67 (−0.34, 0.51)	0.674	−1.2 (−2.0, −0.5)	1.4 (0.7, 2.1)

## Data Availability

The data sets used and/or analyzed are available from the corresponding author on reasonable request.

## Supplementary Materials

The following are available online at https://www.mdpi.com/article/10.3390/ani11113279/s1, Table S1. Demographic and medical data used in the study.
